# Liver-targeted delivery of insulin-loaded nanoparticles *via* enterohepatic circulation of bile acids

**DOI:** 10.1080/10717544.2018.1469685

**Published:** 2018-05-23

**Authors:** Zhe Zhang, Hongxiang Li, Guangrui Xu, Ping Yao

**Affiliations:** State Key Laboratory of Molecular Engineering of Polymers, Department of Macromolecular Science, Collaborative Innovation Center of Polymers and Polymer Composite Materials, Fudan University, Shanghai, China

**Keywords:** Bile acid transporters, chitosan derivative, HPMCP, insulin, oral delivery

## Abstract

Liver is the primary acting site of insulin. In this study, we developed innovative nanoparticles for oral and liver-targeted delivery of insulin by using enterohepatic circulation of bile acids. The nanoparticles were produced from cholic acid and quaternary ammonium modified chitosan derivative and hydroxypropyl methylcellulose phthalate (HPMCP). The nanoparticles had a diameter of 239 nm, an insulin loading efficiency of 90.9%, and a loading capacity of 18.2%. Cell culture studies revealed that the cholic acid groups effectively enhanced the transport of the nanoparticles through Caco-2 cell monolayer and greatly increased the absorption of the nanoparticles in HepG-2 cells *via* bile acid transporter mechanism. *Ex vivo* fluorescence images of ileum section, gastrointestinal tract, and liver demonstrated that the HPMCP increased the mucoadhesion of the nanoparticles in ileum, and the cholic acid groups facilitated the absorptions of the nanoparticles in both ileum and liver by use of bile acid transporters *via* enterohepatic circulation of bile acids. The therapy for diabetic mice displayed that the oral nanoparticle group could maintain hypoglycemic effect for more than 24 h and its pharmacological availability was about 30% compared with the insulin injection group. For the first time, this study demonstrates that using enterohepatic circulation of bile acids is an effective strategy for oral delivery of insulin.

## Introduction

Diabetes mellitus is a worldwide chronic disease and an estimated 415 million people had diabetes worldwide as of 2015 (IDF Diabetes Atalas, 2017). Insulin, secreted by pancreas, is the only hormone in human body that can reduce BGL (blood glucose level) directly (Saltiel & Kahn, 2001, Edgerton et al., 2006). As the primary acting site of insulin, liver plays a major role in carbohydrate metabolism and takes the responsibility for the balance of BGL by means of glycogenogenesis and glycogenolysis (Pessin & Saltiel, 2000; Arbit, 2004; Edgerton et al., 2006). After being transported to liver cells, endogenous insulin stimulates the synthesis of glycogen that transforms the glucose in the blood into the glycogen in the liver and inhibits the breakdown of the glycogen (Pessin & Saltiel, 2000). By now, as the most effective treatment, subcutaneous injection of insulin every day is still the best choice for both type 1 and 2 diabetics (Li et al., 2018). However, subcutaneously injected insulin enters into the general circulation directly, which exposes all tissues to the same insulin concentration and the liver only receives a small fraction of the injected dose; thus, muscles and adipocytes can react to the insulin without hepatic monitoring, and side effects such as atherosclerosis, hypoglycemia and weight gain may occur (Arbit, 2004; Geho et al., 2009).

For insulin therapy, oral administration is most acceptable by diabetics, but the oral bioavailability of insulin is very low due to the physiological barriers in gastrointestinal (GI) tract, including chemical, enzymatic, and absorption barriers (Lopes et al., 2014). The orally administrated insulin undergoes denaturation due to the acidic environment in the stomach and is broken down in the GI tract by the proteolytic enzymes. Furthermore, the absorption of the insulin through the mucus covered intestinal epithelia is limited due to the high molecular weight and hydrophilicity of insulin (Mo et al., 2014). To overcome gastric acid and enzyme damages as well as facilitate the absorption in GI tract, many micro- and nano-sized systems have been fabricated for oral delivery of insulin (Mo et al., 2014). For example, the nanoparticles fabricated from chitosan and its derivatives are mucoadhesive (Zambito et al., 2013), which can enhance the absorption of the loaded insulin in GI tract (Sheng et al., 2016). Hydroxypropyl methylcellulose phthalate (HPMCP), a widely used enteric coating material in pharmaceutical industry (Singh et al., 2015), can protect insulin from degradation and denaturation in the harsh environments of stomach (Du et al., 2015). Makhlof et al. (2011) reported that the acid stability, and the intestinal mucoadhesion and penetration of insulin-loaded chitosan/HPMCP nanoparticles were significantly improved compared with the chitosan/tripolyphosphate nanoparticles.

It was reported that the oral delivery systems conjugated with bile acid can deliver the drugs to the liver directly by use of enterohepatic circulation mechanism (HO, 1987; Swaan et al., 1996; Zhang et al., 2016a). Enterocytes in ileum express apical Na^+^-dependent bile acid transporter (ASBT) and cytosolic ileal bile acid-binding protein (IBABP) (Gong et al., 1994; Kolhatkar & Polli, 2012; Fan et al., 2018). Hepatocytes express bile acid transporters such as Na^+^-dependent taurocholate cotransporting polypeptide (NTCP) and Na^+^-independent organic anion transporting polypeptides (OATPs) (Schadt et al., 2016). These bile acid transporters are involved in the enterohepatic circulation of bile acids. The high capacity of the bile acid transporters and high efficacy of both intestinal and liver absorptions make the enterohepatic circulation of bile acids be utilized in oral delivery systems to increase the therapeutic concentration in the liver and reduce the general toxicity of the drugs (Swaan et al., 1996; Zhang et al., 2016a). For insulin delivery, it was evidenced that the bile acid conjugated insulin was taken up by the ileal bile acid transporters after infusion into the small intestine (McGinn & Morrison, 2016). Very recently, Fan et al. (2018) reported deoxycholic acid-modified nanoparticles produced by self-assembly of insulin, deoxycholic acid-modified chitosan, and poly (γ-glutamic acid). The nanoparticles could overcome multiple barriers of the intestinal epithelium by using ASBT-mediated endocytosis and IBABP-guided intracellular trafficking, and facilitated the basolateral release of free insulin. To the best of our knowledge, no system for oral and liver-targeted delivery of insulin by using enterohepatic circulation of bile acids was reported by now.

To improve oral bioavailability of insulin, in this study, we designed and fabricated a novel delivery system. We used cholic acid and N-(2-hydroxy)-propyl-3-trimethylammonium chloride modified chitosan (HTCC-CA) and HPMCP to produce insulin-loaded nanoparticles INS/HTCC-CA/HPMCP. The nanoparticles were expected to prevent the loaded insulin from denaturation and degradation in GI tract as well as to improve the intestinal mucoadhesion. The nanoparticles were also expected to facilitate the intestinal and liver absorptions of the loaded insulin by utilizing the enterohepatic circulation of bile acids. We performed a series of experiments to reveal the absorption mechanism of the nanoparticles in intestinal and liver, and to prove that the nanoparticles can greatly improve the oral bioavailability of insulin.

## Materials and methods

### Materials

Chitosan (50 kDa, deacetylation degree 85%) was purchased from Jinan Haidebei Marine Bioengineering Co., Ltd, Shandong, China. Insulin (INS, 21 IU/mg) was from Dingguo Biotech Co., Ltd, Shanghai, China. HPMCP (HP-55S) was from Huzhou Mizuda Hope Bioscience Co., Ltd, Huzhou, China. Alloxan was from Sigma-Aldrich (Chicago, IL). Fluorescein isothiocyanate (FITC) and Rhodamine B (RhB) were from Tokyo Chemical Industry Co., Ltd, Tokyo, Japan. Sulfo-Cyanine5 NHS ester (Cy5) was from Lumiprobe, Hunt Valley, MD. Bicinchoninic acid (BCA) protein assay kit was from Thermo Fisher Scientific Inc, Waltham, MA. DMEM, fetal bovine serum, MEM non-essential amino acid solution, L-glutamine, penicillin and streptomycin were from GIBCO BRL Life Technologies Inc., Carlsbad, CA. 3-(4,5-Dimethylthiazol-2-yl)-5-(3-carboxymethoxyphenyl)-2-(4-sulfophenyl)-2*H*-tetrazolium (MTS) was from Promega Co., Madison, WI. 2-(4-Amidinophenyl)-6-indolecarbamidine dihydrochloride (DAPI) was from Beyotime Institute of Biotechnology, Jiangsu, China. DAPI Fluoromount-G^TM^ was from Yeasen Biotechnology, Shanghai, China. All other chemicals were of analytical grade and from Sinopharm Chemical Reagent Co., Ltd, Shanghai, China.

### Preparation of nanoparticles

HTCC (N-(2-hydroxy)-propyl-3-trimethylammonium chloride modified chitosan) and HTCC-CA (cholic acid modified HTCC) were synthesized, purified, and characterized as reported previously (Zhang et al., 2016b). The quaternary ammonium degree was 35.8% and CA (cholic acid) conjugation degree was 5.7% of the glycosyl units of chitosan. HTCC, HTCC-CA, and HPMCP stock solutions were prepared by dissolving the polymers in deionized water respectively and adjusting the solutions to pH 7.4. Insulin stock solution was prepared by dissolving insulin in 0.01 M HCl solution and adjusting the solution to pH 7.4.

INS/HTCC-CA nanoparticles were prepared by dropwise adding 1 mL of 1 mg/mL insulin solution into 2 mL of 1 mg/mL HTCC-CA solution with gentle stir. Successively, 3 mL of the INS/HTCC-CA nanoparticle solution was added dropwise into 2 mL of 1 mg/mL HPMCP solution with gentle stir to produce INS/HTCC-CA/HPMCP nanoparticles. Similarly, INS/HTCC/HPMCP nanoparticles were prepared. The final concentrations of insulin, HTCC or HTCC-CA, and HPMCP in INS/HTCC/HPMCP and INS/HTCC-CA/HPMCP nanoparticles were 0.2, 0.4, and 0.4 mg/mL, respectively. Both the final concentrations of insulin and HTCC-CA in INS/HTCC-CA nanoparticles were 0.2 mg/mL.

### Characterization of the nanoparticles

Z-Average hydrodynamic diameter (D_h_), polydispersity index (PDI), and ζ-potential of the nanoparticles were measured on a laser light scattering instrument (Zetasizer Nano ZS90, Malvern Instruments, Malvern, UK) as reported previously (Zhang et al., 2016b). Transmission electron microscopy (TEM) images of the nanoparticles were acquired on a transmission electron microscope (Philips CM120 electron microscope, Philips, Amsterdam, Netherlands). Free insulin in the nanoparticle solutions was separated using centrifugal filter (cutoff molecular weight 100 kDa, Millipore, Billerica, MA) and the insulin concentrations in the filtrates were analyzed using BCA assay. The insulin loading efficiency (LE) and loading capacity (LC) of the nanoparticles were calculated using the following equations:

$$LE\;(\%,\;\frac{wt}{wt})=\frac{\mathrm{total}\;\mathrm{insulin}-\mathrm{free}\;\mathrm{insulin}}{\mathrm{total}\;\mathrm{insulin}}\times 100\%$$

$$LC\;(\%,\;\frac{wt}{wt})=\frac{\mathrm{total}\;\mathrm{insulin}-\mathrm{free}\;\mathrm{insulin}}{\mathrm{total}\;\mathrm{polymers}+\mathrm{total}\;\mathrm{insulin}}\times 100\%$$

## In vitro *release of insulin from the nanoparticles*

Insulin releases from the nanoparticles were investigated by dialysis of 1 mL of the nanoparticle solution (cutoff molecular weight 100 kDa, Spectrum Laboratories Inc., Piscataway Township, NJ) against 4 mL of pH 2.0 HCl solution or pH 7.4 PBS (0.01 M phosphate buffer containing 0.15 M NaCl) solution at 37 °C with shaking. At predetermined intervals, 1 mL of the release medium was taken out and the same volume of fresh medium was added. The insulin concentration in the release medium was determined using BCA assay.

### Preparation of fluorescence-labeled nanoparticles

FITC-labeled insulin (FITC-INS) and Cy5-labeled insulin (Cy5-INS) were synthesized and purified as described in the literature (Wang et al., 2016; Zhang et al., 2016b). The fluorescence-labeled nanoparticles were prepared as described above using FITC-INS or Cy5-INS instead of insulin. Similarly, RhB-labeled HTCC (RhB-HTCC), RhB-labeled HTCC-CA (RhB-HTCC-CA), and FITC-labeled HPMCP (FITC-HPMCP) were synthesized and purified, and Cy5-INS/RhB-HTCC-CA, Cy5-INS/RhB-HTCC/FITC-HPMCP and Cy5-INS/RhB-HTCC-CA/FITC-HPMCP nanoparticles were produced.

### Permeability of the nanoparticles across Caco-2 cell monolayer

Caco-2 cells were seeded in transwell inserts at a density of 5 × 10^3^ cells/well and were cultured as reported in the literature (Sheng et al., 2016) for 14 – 21 d until their trans-epithelial electrical resistance (TEER) values were higher than 900 Ω·cm^2^. The cell monolayer was washed with PBS thrice, then 0.2 mL DMEM containing insulin or insulin-loaded nanoparticles with insulin concentration of 50 μg/mL, or containing individual polymer with the concentration of 100 μg/mL, or containing free CA molecules with the concentration of 100 μM was added into the apical side; 0.6 mL DMEM without sample was added into the basolateral side. After 1 h incubation, the medium was removed. The cell monolayer was washed with PBS thrice and then was cultured in fresh DMEM for 9 h. At predetermined intervals, TEER of the Caco-2 cell monolayer was measured using an electrical resistance system (ERS-2, Millipore, Billerica, MA).

Apparent permeability coefficient (*P*_app_) of insulin was measured as follows. After washing the Caco-2 cell monolayer with PBS, 0.2 mL DMEM containing FITC-INS or FITC-INS loaded nanoparticles with insulin concentration of 50 μg/mL was added into the apical side; 0.6 mL DMEM was added into the basolateral side. At predetermined intervals, sample was collected from the basolateral side and the same volume of fresh DMEM was added. The FITC-INS concentration in the sample was measured on a fluorescence microplate reader (Cytation3, BioTek, Winooski, VT). The *P*_app_ of insulin was calculated using the following equation:

$$P_{app}=\frac{Q}{Act}$$

where *Q* is the total amount of insulin permeated (ng), *A* is the diffusion area of the cell monolayer (cm^2^), *c* is the initial concentration of insulin in the donor compartment (ng/cm^3^), and *t* is the total time of the experiment (s).

### Cellular uptake of insulin by HepG-2 cells

HepG-2 cells were seeded in special Petri dishes at a density of 1 × 10^5^ cells/well and cultured as reported previously (Zhang et al., 2016b). Subsequently, the cells were incubated with the culture medium containing FITC-INS or FITC-INS-loaded nanoparticles at insulin concentration of 50 μg/mL. After 4 h incubation, the cells were washed with PBS thrice and the cell nuclei were stained with DAPI for 5 min, and then the cells were observed on a confocal laser scanning microscope (CLSM, C2+, Nikon, Tokyo, Japan). The cellular uptakes of insulin were determined quantitatively using flow cytometry analysis. After 4 h incubation with the culture medium containing FITC-INS or FITC-INS loaded nanoparticles at insulin concentration of 50 μg/mL, the cells were washed with PBS thrice and then analyzed on a flow cytometer (FACSCalibur, BD, Franklin Lakes, NJ).

## In vivo *biocompatibility*

Female ICR mice (25 ± 2 g) were from Sino-British SIPPR/BK Lab Animal Ltd, Shanghai, China. The animal experiments of this study were performed at Experimental Animal Center of School of Pharmacy of Fudan University in full compliance with the guidelines approved by Shanghai Administration of Experimental Animals.

Healthy mice were separately administrated by gastric gavage with 0.2 mL mixed solution of HTCC-CA (10 mg/mL) and HPMCP (10 mg/mL) once daily for 15 and 30 d continuously. After the mice were sacrificed, the organ sections were prepared as reported previously (Zhang et al., 2016b). Histological images of the organ sections were acquired on a microscope (BX53, OLYMPUS, Tokyo, Japan).

## Ex vivo *fluorescence imaging of ileum section*

Healthy mice were fasting for 12 h with freedom to water. Cy5-INS/RhB-HTCC-CA, Cy5-INS/RhB-HTCC/FITC-HPMCP, and Cy5-INS/RhB-HTCC-CA/FITC-HPMCP nanoparticles were separately administrated by gastric gavage at insulin dose of 30 IU/kg. The mice were sacrificed after 4 h of the administration. The ileum segments were taken out and washed with PBS thrice. The ileum segments were frozen in cryoembedding medium followed by cryostat section. The section was loaded on a microscope slide and fixed with DAPI fluoromount-G^TM^. The images of the section were acquired on the CLSM.

## Ex vivo *fluorescence imaging of organs*

Healthy mice were fasting for 12 h with freedom to water. Cy5-INS/HTCC-CA, Cy5-INS/HTCC/HPMCP, and Cy5-INS/HTCC-CA/HPMCP nanoparticles were orally administrated at insulin dose of 30 IU/kg. The mice were sacrificed at 0, 2, 6, 12, and 24 h post-administration. The organs were excised and washed with PBS thrice. *Ex vivo* fluorescence images of the organs were observed on a small animal imaging system (In Vivo Xtreme, Bruker, Billerica, MA) and the sum fluorescence intensities of the organs were measured.

### Antidiabetic efficacy

Healthy mice were intraperitoneally injected with alloxan solution at a single dose of 200 mg/kg to induce type 1 diabetes as reported previously (Zhang et al., 2016b). The blood from caudal vein was sampled and the BGL was measured using a glucometer (ACCUCHEK Active, Roche). The diabetic mice with average fasting BGL of 21.7 ± 3.5 mM were divided into five groups with five in each group. The mice were fasting for 10 h with freedom to water prior to administration. Insulin solution was injected subcutaneously into the mice at insulin dose of 3 IU/kg. Physiological saline, INS/HTCC-CA, INS/HTCC/HPMCP, and INS/HTCC-CA/HPMCP nanoparticles were separately administrated by gastric gavage at an insulin dose of 30 IU/kg. At predetermined intervals, the BGL was measured. At 4 h post-administration, about 0.2 g standard chow was provided for each of the mice. Insulin pharmacological availability (PA) of the nanoparticle (NP) groups were calculated according to the area above the relative BGL-time curve (AAC) using the following equation:

$$PA\;\left(\%\right)=\frac{({AAC}_{NP,\;ig}-{AAC}_{saline})/{Dose}_{NP,\;ig}}{{(AAC}_{INS,\;sc}-{AAC}_{saline})/{Dose}_{INS,\;sc}}\times 100\%$$

For repeated administrations, diabetic mice with average fasting BGL of 17.5 ± 7.6 mM were divided into four groups with five in each group. Insulin solution was injected subcutaneously at insulin dose of 2 IU/kg once daily. Physiological saline, insulin solution, and INS/HTCC-CA/HPMCP nanoparticles were separately administrated by gastric gavage at insulin dose of 30 IU/kg once daily. Rat chow was provided at 6 – 12 h post-administration. Water was provided at all times. During the experiment, two mice in the insulin injection group and one mouse in the INS/HTCC-CA/HPMCP oral group were died of hypoglycemia.

### Statistical analysis

The data were expressed as mean ± SD (standard deviation). Statistical analysis was performed using independent samples-*t* test (OriginPro 8.0 software, SAS Inc., Cary, NC), and a *p* value <.05 was considered to be statistically significant.

## Results and discussion

### Preparation and characterization of insulin-loaded nanoparticles

**Figure 1.**
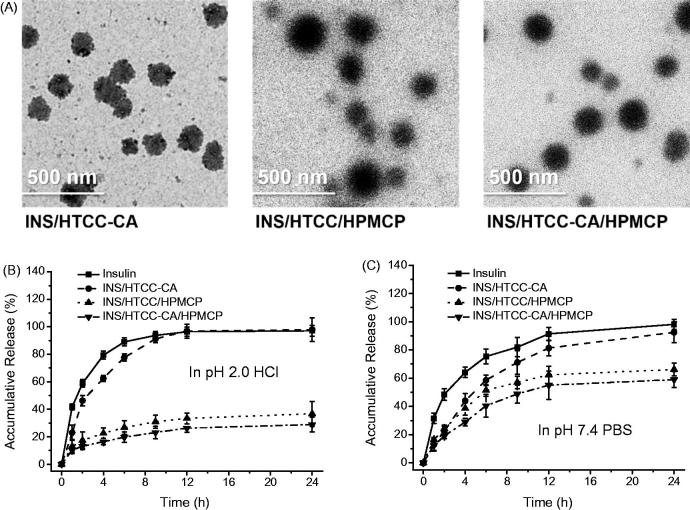
(A) TEM images of the nanoparticles. (B) and (C) *In vitro* accumulative releases of insulin from the nanoparticles in pH 2.0 HCl and pH 7.4 PBS solutions at 37 °C (*n* = 3); individual insulin solution was assayed as the control.

INS/HTCC-CA/HPMCP nanoparticles were prepared after mixing insulin with HTCC-CA and then HPMCP in pH 7.4 solution by means of electrostatic and hydrophobic interactions. For comparison, INS/HTCC/HPMCP and INS/HTCC-CA nanoparticles were prepared using the same process. In pH 7.4 solution, INS/HTCC-CA had D_h_ and ζ-potential of 168 nm and 19.5 mV, respectively, as shown in Table S1 of Supplemental data. The *D*_h_ and ζ-potential of INS/HTCC-CA/HPMCP were 239 nm and –24.2 mV, respectively. The ζ-potential of INS/HTCC-CA/HPMCP was less negative than the ζ-potential of HPMCP (–29.2 ± 0.12 mV) at pH 7.4 condition, indicating that the surface of INS/HTCC-CA/HPMCP was composed of both positively charged HTCC-CA and negatively charged HPMCP. INS/HTCC/HPMCP had similar ζ-potential to INS/HTCC-CA/HPMCP, but the *D*_h_ of INS/HTCC/HPMCP was about 55 nm larger than that of INS/HTCC-CA/HPMCP. Possibly, INS/HTCC-CA/HPMCP had more compact structure than INS/HTCC/HPMCP due to the hydrophobic interaction introduced by the CA groups. Figure 1(A) shows TEM images of INS/HTCC-CA, INS/HTCC/HPMCP, and INS/HTCC-CA/HPMCP. The nanoparticles presented globular morphology, and their sizes shown in the images were similar to the *D*_h_ values. The LE values of INS/HTCC-CA, INS/HTCC/HPMCP, and INS/HTCC-CA/HPMCP were 96.7%, 87.8%, and 90.9%, respectively (Table S1 of Supplemental data), indicating that all the three systems can effectively encapsulate insulin.

**Table 1.** Pharmacodynamics parameters after single subcutaneous injection of free insulin solution and gastric gavage with saline and the nanoparticles (*n* = 5).

Group	INS dose (IU/kg)	Method	BGL_min_ (%)	PT-70% (h)	AAC_0-24_^a^	PA (%)
Insulin	3	s.c.	37.2	2.7	304.0	100
INS/HTCC-CA	30	i.g.	75.6	–	220.9	7.3
INS/HTCC/HPMCP	30	i.g.	71.9	–	341.2	11.2
INS/HTCC-CA/HPMCP	30	i.g.	54.1	20.0	817.9	26.9

a The area above the curve shown in Figure 5(A) during 0–24 h.

## In vitro *insulin release*

Insulin releases from the nanoparticles were investigated using a dialysis method in pH 2.0 HCl and pH 7.4 PBS media to mimic the pH environments in stomach and intestine. Figure 1(B) shows that at pH 2.0 condition, the insulin release curve of INS/HTCC-CA was similar to the insulin diffusion curve of individual insulin solution, suggesting that INS/HTCC-CA dissociated rapidly in pH 2.0 solution. Both insulin and HTCC-CA were positively charged at pH 2.0, thus the electrostatic repulsion resulted in the rapid dissociation of INS/HTCC-CA. INS/HTCC/HPMCP and INS/HTCC-CA/HPMCP were stable at pH 2.0; only about 20% of the insulin was released from the nanoparticles in the first 6 h. It was reported that HPMCP can protect the loaded drugs in acidic environment by solution–microgel transition as a result of the protonation of the carboxyl groups (Singh et al., 2015). At acidic condition, the protonated HPMCP made INS/HTCC/HPMCP and INS/HTCC-CA/HPMCP more stable that reduced their insulin release rates. In pH 7.4 PBS solution, INS/HTCC-CA was also unstable and released most of the insulin after 24 h because the salt in PBS shielded the electrostatic interaction between insulin and HTCC-CA. The insulin release rates of INS/HTCC/HPMCP and INS/HTCC-CA/HPMCP were slower than the rate of INS/HTCC-CA, demonstrating that HPMCP increased the stability of the nanoparticles. In addition, the insulin release rates of INS/HTCC-CA/HPMCP were slower than the rates of INS/HTCC/HPMCP in both pH 2.0 and 7.4 media, indicating that the hydrophobic interaction introduced by the CA groups increased the stability of the nanoparticles.

### Transport through Caco-2 cell monolayer

**Figure 2.**
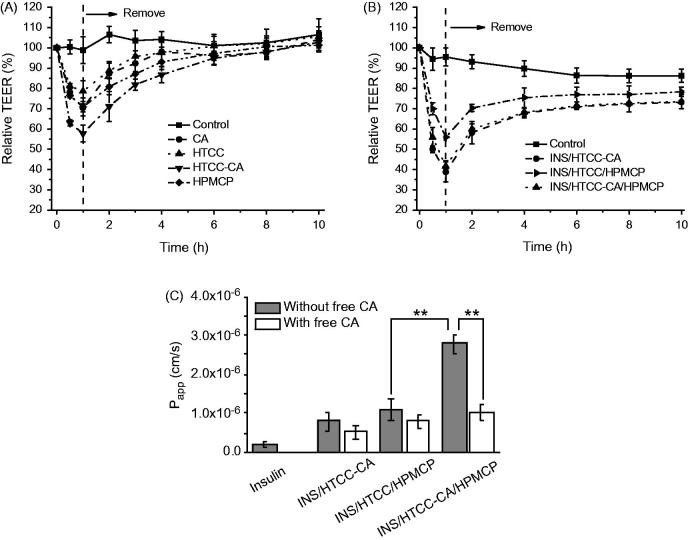
Relative TEER changes of Caco-2 cell monolayers after incubation then remove of (A) free CA (100 μM) and the individual polymer (100 μg/mL), and (B) the nanoparticles with insulin concentration of 50 μg/mL; (C) apparent permeability coefficients of FITC-INS in Caco-2 cell monolayers after incubation with the FITC-INS loaded nanoparticles at insulin concentration of 50 μg/mL; the monolayers were pretreated with 100 μM free CA molecules for 30 min or not (*n* = 3). **p < .01.

Caco-2 cells express bile acid transporters ASBT and IBABP, and Caco-2 cell monolayer can be used to mimic the enterocytes in studying the transport of the delivery system in intestinal barrier *in vitro* (Alam et al., 2014; Fan et al., 2018). The decrease of TEER value is considered as an open indication of the tight junctions between Caco-2 cells (Hsu et al., 2013). All the three polymers as well as individual CA reduced the TEER values significantly as shown in Figure 2(A). HTCC-CA had stronger impact on the TEER change than the others. The TEER changes were reversible after remove of the samples, suggesting that the cell monolayers recovered their integrity gradually. Figure 2(B) shows that INS/HTCC-CA and INS/HTCC-CA/HPMCP groups had similar TEER changes, and both INS/HTCC-CA and INS/HTCC-CA/HPMCP groups reduced the TEER values more than the INS/HTCC/HPMCP group.

Figure 2(C) shows *P*_app_ values of FITC-INS through Caco-2 cell monolayers after incubation with free FITC-INS and FITC-INS loaded nanoparticles. The permeability of free FITC-INS in the monolayer was poor as indicated by its *P*_app_ value of 2 × 10^−7 ^cm/s. All the three FITC-INS-loaded nanoparticles facilitated the permeation of the FITC-INS through the monolayers. The *P*_app_ value of the FITC-INS/HTCC-CA/HPMCP group was 2.8 × 10^−6 ^cm/s, which was 2.5-fold higher than that of the FITC-INS/HTCC/HPMCP group, indicating that FITC-INS/HTCC-CA/HPMCP transported the loaded FITC-INS through the Caco-2 cell monolayer *via* the bile acid transporters, ASBT-mediated endocytosis and IBABP-guided intracellular trafficking as reported in the literature (Fan et al., 2018). To further prove this transport mechanism, the cell monolayers were pretreated with 100 μM free CA molecules for 30 min to block the bile acid transporters as reported in the literature (Khatun et al., 2014). Although free CA induced perturbation in the cell monolayer (Figure 2(A)), in the presence of free CA, the *P*_app_ value of the FITC-INS/HTCC-CA/HPMCP group was very low, which was almost the same as the value of the FITC-INS/HTCC/HPMCP group (Figure 2(C)). This result reveals that most of the FITC-INS/HTCC-CA/HPMCP could not transport the loaded FITC-INS through the Caco-2 cell monolayer when the bile acid transporters were blocked by free CA molecules. This result confirms that the CA groups on FITC-INS/HTCC-CA/HPMCP surface could bind with the bile acid transporters, and by means of the bile acid transporters, FITC-INS/HTCC-CA/HPMCP greatly enhanced the transport of the FITC-INS through Caco-2 cell monolayer.

INS/HTCC-CA also had CA groups on the surface, and the INS/HTCC-CA group had similar TEER change to the INS/HTCC-CA/HPMCP group. However, the *P*_app_ value of the FITC-INS/HTCC-CA group was only 30% of the *P*_app_ value of the FITC-INS/HTCC-CA/HPMCP group. Pretreating the Caco-2 cell monolayer with free CA molecules had no great influence on the *P*_app_ of the FITC-INS/HTCC-CA group, suggesting that most of the FITC-INS/HTCC-CA could not go through the monolayer *via* the bile acid transporters. This result can be explained by the facts that INS/HTCC-CA was unstable as proved in Figure 1(B, C) and the released insulin had very low *P*_app_ value as demonstrated in Figure 2(C).

### HepG-2 cellular uptake

**Figure 3.**
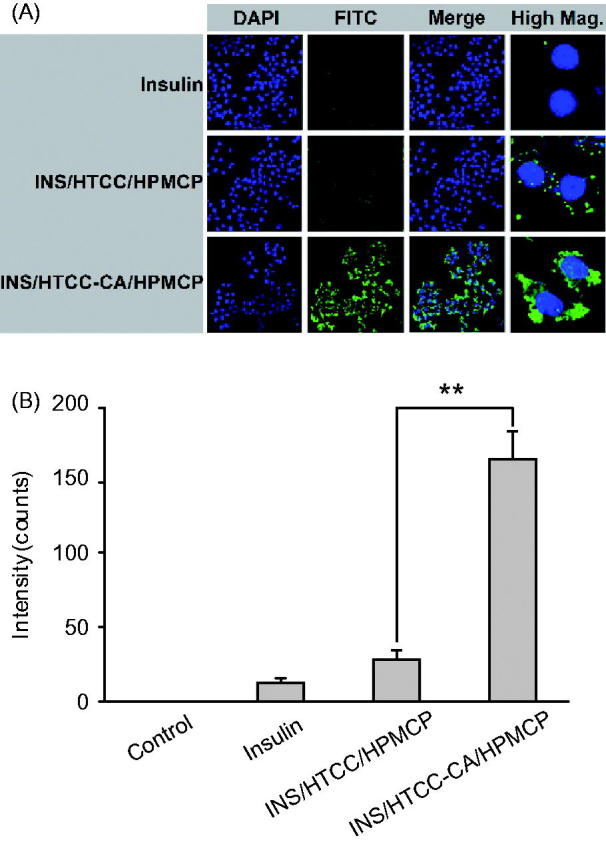
HepG-2 cellular uptakes after 4 h incubation with FITC-INS, FITC-INS/HTCC/HPMCP, and FITC-INS/HTCC-CA/HPMCP at insulin concentration of 50 μg/mL. (A) CLSM images of HepG-2 cells, and (B) geometric mean values of FITC-INS fluorescence intensities of the flow cytometry analysis (*n* = 3). The cell nuclei were stained with DAPI. **p < .01.

Liver is the primary acting site of insulin (Arbit, 2004). HepG-2 cells express bile acid transporters, such as NTCP and OATPs (Swaan et al., 1996; Zhang et al., 2016a). Therefore, in this study, HepG-2 cells were used as an *in vitro* hepatocyte model to investigate hepatocyte uptake of the nanoparticles. Figure 3(A) shows CLSM images of HepG-2 cells after 4 h incubation with free FITC-INS, FITC-INS/HTCC/HPMCP, and FITC-INS/HTCC-CA/HPMCP at insulin concentration of 50 μg/mL. The images show that the FITC-INS/HTCC-CA/HPMCP group had much stronger FITC-INS fluorescence signal around the cell nuclei than the other groups. The cellular uptakes of FITC-INS were quantificationally characterized by flow cytometry analysis. Figure 3(B) shows that the FITC-INS fluorescence intensities of FITC-INS/HTCC/HPMCP and FITC-INS/HTCC-CA/HPMCP groups were 1.4-fold and 8.5-fold higher than the intensity of the free FITC-INS group, respectively, confirming that the CA groups on FITC-INS/HTCC-CA/HPMCP surface can facilitate the cellular uptake by means of the bile acid transporters.

## In vivo *biocompatibility*

Figure S1 of Supplemental data shows hematoxylin − eosin-stained histological images of heart, liver, spleen, lung, kidney, stomach, and intestine of the mice after oral administration with HTCC-CA and HPMCP. The daily polymer dose in biocompatibility study was 30-fold higher than the dose of INS/HTCC-CA/HPMCP in the hypoglycemic study. Compared with the control group which was denoted as 0 d in Figure S1, the polymers did not induce significant morphological changes in the tissues after 15 and 30 d of continuous administrations, verifying that HTCC-CA and HPMCP, the carriers of insulin in this study, were biocompatible.

### Distribution of the nanoparticles in histological section of ileum

**Figure 4.**
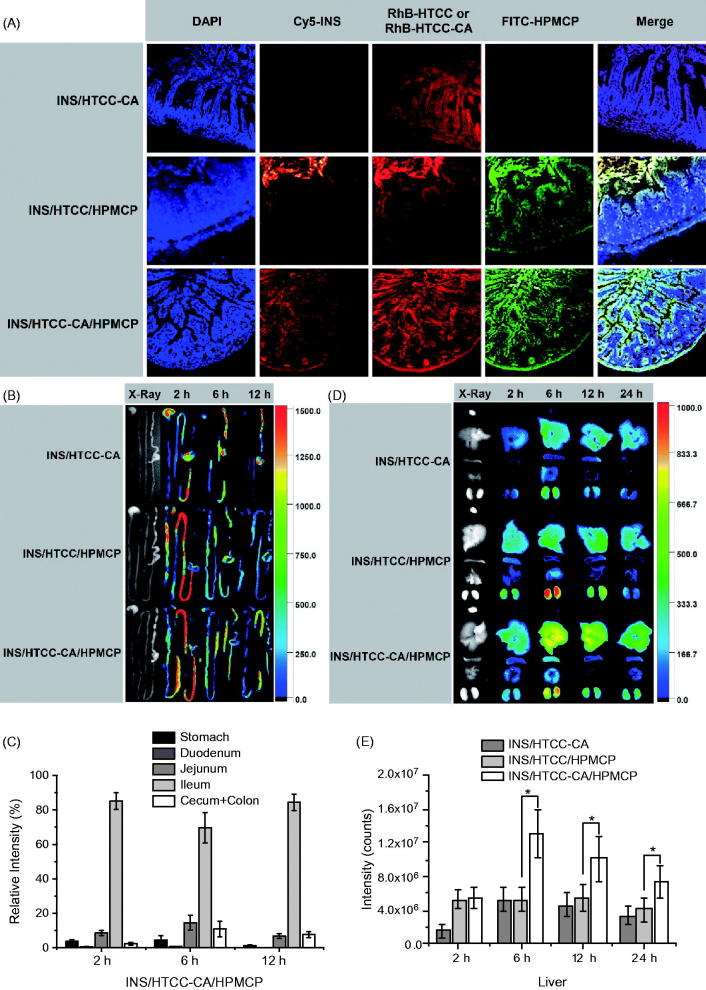
(A) CLSM images of ileum sections of the mice after oral administrations with Cy5-INS/RhB-HTCC-CA, Cy5-INS/RhB-HTCC/FITC-HPMCP, and Cy5-INS/RhB-HTCC-CA/FITC-HPMCP; the ileums were excised at 4 h post-administration. (B) Representative fluorescence images of the GI tracts excised at 0, 2, 6, and 12 h after oral administrations with Cy5-INS-loaded nanoparticles and (C) relative fluorescence intensity distributions of Cy5-INS/HTCC-CA/HPMCP in the GI tracts. (D) Representative fluorescence images of heart, liver, spleen, lung, and kidney excised at 0, 2, 6, 12 and 24 h after oral administrations with Cy5-INS loaded nanoparticles and (E) fluorescence intensities of liver after oral administrations with Cy5-INS loaded nanoparticles. The insulin dose was 30 IU/kg, *n* = 3, and *p < .05.

Figure 4(A) shows the CLSM images of ileum sections of the mice after oral administrations with Cy5-INS/RhB-HTCC-CA, Cy5-INS/RhB-HTCC/FITC-HPMCP, and Cy5-INS/RhB-HTCC-CA/FITC-HPMCP at insulin dose of 30 IU/kg for 4 h. For the Cy5-INS/RhB-HTCC-CA group, only RhB-HTCC-CA was detected, indicating that Cy5-INS/RhB-HTCC-CA dissociated in the GI tract and the released Cy5-INS could not be absorbed by the epithelium. For the Cy5-INS/RhB-HTCC/FITC-HPMCP group, Cy5-INS as well as RhB-HTCC and FITC-HPMCP were mainly distributed in the mucous layer. This result reveals that INS/HTCC/HPMCP was stable in the GI tract and prolonged the stay in the mucous layer, but could not be absorbed effectively. For the Cy5-INS/RhB-HTCC-CA/FITC-HPMCP group, no significant accumulation in the mucous layer, Cy5-INS/RhB-HTCC-CA/FITC-HPMCP was mainly distributed in the epithelium, demonstrating that Cy5-INS/RhB-HTCC-CA/FITC-HPMCP was absorbed effectively by the epithelium *via* bile acid transporters.

### Distribution of the nanoparticles in GI tract

Figure 4(B) shows fluorescence images of stomachs and intestines of the mice after oral administrations with Cy5-INS loaded nanoparticles at insulin dose of 30 IU/kg. At 2 h post-administration, the total fluorescence intensity of the Cy5-INS/HTCC-CA/HPMCP group was lower than the intensities of the Cy5-INS/HTCC-CA group and the Cy5-INS/HTCC/HPMCP group as shown in Figure S2 of Supplemental data. This result indicates that more Cy5-INS/HTCC-CA/HPMCP was absorbed in the GI tract, and this conclusion is supported by the result shown in Figure 4(A). Therefore, at each time interval (2, 6, and 12 h post-administration), relative fluorescence intensities of the gastrointestinal segments are shown in Figure 4(C) for the Cy5-INS/HTCC-CA/HPMCP group and in Figure S3 (Supplemental data) for the other groups to characterize the distributions of the nanoparticles in the GI tracts. For the Cy5-INS/HTCC-CA/HPMCP group, more than 70% of the nanoparticles retained in ileum at 2, 6, and 12 h post-administration, higher than the ileum distributions of Cy5-INS/HTCC-CA and Cy5-INS/HTCC/HPMCP groups. The increase of the ileum retention provides higher probability for Cy5-INS/HTCC-CA/HPMCP to contact with the epithelium and to bind with the bile acid transporters, and thus higher probability to go through the intestinal epithelium.

### Distribution of the nanoparticles in organs

Figure 4(D) shows fluorescence images of heart, liver, spleen, lung, and kidney of the mice after oral administrations with Cy5-INS-loaded nanoparticles at insulin dose of 30 IU/kg. At each time interval (2, 6, 12, and 24 h post-administration), the total fluorescence intensity of the Cy5-INS/HTCC-CA/HPMCP group was higher than the intensities of the other groups (Figure S4 of Supplemental data), confirming that more Cy5-INS/HTCC-CA/HPMCP was absorbed in the GI tract, subsequently more Cy5-INS/HTCC-CA/HPMCP was accumulated in the organs. The Cy5-INS/HTCC-CA/HPMCP group had higher Cy5-INS fluorescence intensity in the liver and retained the Cy5-INS in the liver for longer time than the other groups as shown in Figure 4(E). Especially, after oral administration, the Cy5-INS/HTCC-CA/HPMCP group accumulated more Cy5-INS in the liver for longer time than the Cy5-INS/HTCC/HPMCP group, further confirming that the CA groups of Cy5-INS/HTCC-CA/HPMCP facilitated the liver uptake of the nanoparticles by means of the bile acid transporters.

### Hypoglycemic effect of the nanoparticles

**Figure 5.**
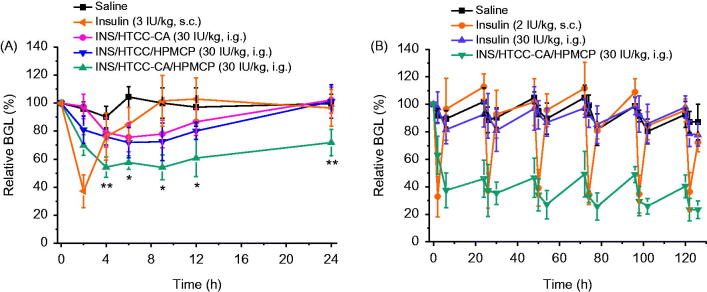
(A) Relative BGL changes of diabetic mice after single subcutaneous injection of free insulin solution at insulin dose of 3 IU/kg and gastric gavage with saline and the nanoparticles at insulin dose of 30 IU/kg (*n* = 5); *p < .05 and **p < .01 between INS/HTCC/HPMCP and INS/HTCC-CA/HPMCP groups. (B) Relative BGL changes of diabetic mice after subcutaneous injection of insulin at a dose of 2 IU/kg and oral administrations with saline, insulin, and INS/HTCC-CA/HPMCP at insulin dose of 30 IU/kg once daily for 5 d continually (*n* = 3 – 5).

Figure 5(A) shows the relative BGL changes of diabetic mice after administrations with free insulin and the nanoparticles. The pharmacodynamics parameters are shown in Table 1. The subcutaneous injection group had good hypoglycemic effect at insulin dose of 3 IU/kg, but the BGL quickly returned to the level of the saline group after reaching the minimum BGL value (BGL_min_), 37.2% of the initial level. INS/HTCC-CA, INS/HTCC/HPMCP, and INS/HTCC-CA/HPMCP were orally administrated at single insulin dose of 30 IU/kg separately. Although INS/HTCC-CA was not stable in GI tract and could not prolong the retention in ileum as demonstrated above, the INS/HTCC-CA group had some hypoglycemic effect: the BGL_min_ was 75.6% and the relative pharmacological availability of insulin (PA) was 7.3% compared with the injection group. The PA of the INS/HTCC-CA/HPMCP group was 26.9%, much higher than the PA of 11.2% of the INS/HTCC/HPMCP group, clearly demonstrating that INS/HTCC-CA/HPMCP was superior to INS/HTCC/HPMCP. Compared with the injection group, the INS/HTCC-CA/HPMCP group had rapid, mild, and lasting hypoglycemic effect: the BGL at 2 h post-administration was 70.0% of the initial level, BGL_min_ was 54.1%, and the time period of the BGL lower than 70% (PT-70%) was 20 h.

Continuous hypoglycemic effects were evaluated by oral administrations with INS/HTCC-CA/HPMCP and free insulin once daily at insulin dose of 30 IU/kg for 5 d continually. Subcutaneous injection of free insulin solution once daily at insulin dose of 2 IU/kg was performed as the control. The oral insulin group had no significant hypoglycemic effect compared with the saline group (Figure 5(B)), which is the same as reported in the literature (Chuang et al., 2015). The injection group had rapid and short-acting effect after each administration of free insulin, the BGL reduced to about 40% of the initial level and recovered within 6 h. The oral INS/HTCC-CA/HPMCP group had stable hypoglycemic effect, the BGL was kept at a steadily low level during the treatment, and the BGL value was lower than 50% of the initial level at each 24 h post-administration. The PA of the oral INS/HTCC-CA/HPMCP group was 36.7% compared with the injection group. The result in Figure 5(B) further confirms that INS/HTCC-CA/HPMCP had prolonged and effective hypoglycemic effect after oral administration.

Oral delivery of insulin has been studied for many years, but the onset time, BGL control and PA of oral insulin were not satisfied yet, and no delivery system was applicable (Mo et al., 2014). As a hydrophilic polypeptide, the orally administrated insulin undergoes multiple biological barriers (Lopes et al., 2014). Stable nanoparticles can protect the insulin from denaturation and degradation in GI tract (Lopes et al., 2014; Fan et al., 2018). In this study, INS/HTCC-CA/HPMCP was more stable than INS/HTCC-CA and INS/HTCC/HPMCP as shown in Figure 1(B, C). Therefore, INS/HTCC-CA/HPMCP protected the loaded insulin in GI tract better than the others. The results in Figure 4 demonstrate that the INS/HTCC-CA/HPMCP group had higher ileum distribution than the other groups that increased the interaction of INS/HTCC-CA/HPMCP with the epithelium. Most importantly, the enterohepatic circulation of bile acids was utilized in this study to deliver the loaded insulin to liver. As demonstrated by the results shown in Figures 2–4, INS/HTCC-CA/HPMCP utilized ASBT-mediated endocytosis, IBABP-guided intracellular trafficking and NTCP/OATPs-mediated endocytosis to go through the epithelium, reach the liver, and be internalized by the hepatocytes. As mentioned above, liver is the primary acting site of insulin. INS/HTCC-CA/HPMCP increased accumulation and prolonged retention time of the insulin in the liver; therefore, INS/HTCC-CA/HPMCP had much better hypoglycemic effect than the other nanoparticles as shown in Figure 5 and Table 1. For the first time, this study demonstrates that using enterohepatic circulation of bile acids can effectively deliver the loaded insulin to liver.

## Conclusions

In this study, we developed innovative INS/HTCC-CA/HPMCP nanoparticles for oral and liver-targeted delivery of insulin. This is the first study of using enterohepatic circulation of bile acids to deliver the loaded insulin to liver after oral administration. This study demonstrates that INS/HTCC-CA/HPMCP protected the loaded insulin from denaturation and degradation in GI tract, the HPMCP increased the mucoadhesion of INS/HTCC-CA/HPMCP in ileum, and the CA groups greatly enhanced the absorptions of INS/HTCC-CA/HPMCP in both ileum and liver. INS/HTCC-CA/HPMCP increased oral PA of the loaded insulin to about 30% and could maintain hypoglycemic effect for more than 24 h. This study demonstrates that using enterohepatic circulation of bile acids to deliver the loaded insulin to liver is an effective strategy for oral insulin delivery, and HTCC-CA/HPMCP is a suitable carrier for oral insulin delivery.

## Supplementary Material

Supplemental Material
