# Supplementary material for: Liver-targeted delivery of insulin-loaded nanoparticles via enterohepatic circulation of bile acids
Source: Drug Deliv. 2018 May 23;25(1):1224–33. doi: 10.1080/10717544.2018.1469685 (PMC6058709; doi:10.1080/10717544.2018.1469685)
Supplement: Supplemental Material [file IDRD_A_1469685_SM3189.docx]

**Supplemental data**

**Table S1.** Size distribution, ζ-potential, LE and LC of the nanoparticles in pH 7.4 aqueous solutions (n = 3).

| Sample | D_h_ (nm) |  | PDI | ζ-potential (mV) | LE (%) | LC (%) |
| --- | --- | --- | --- | --- | --- | --- |
| INS/HTCC-CA | 168 ± 13 |  | 0.23 ± 0.02 | 19.5 ± 1.0 | 96.7 ± 2.1 | 48.4 ± 2.1 |
| INS/HTCC/HPMCP | 294 ± 6 |  | 0.12 ± 0.03 | −26.7 ± 0.9 | 87.8 ± 0.9 | 17.6 ± 0.2 |
| INS/HTCC-CA/HPMCP | 239 ± 4 |  | 0.12 ± 0.02 | −24.2 ± 1.2 | 90.9 ± 1.6 | 18.2 ± 0.4 |


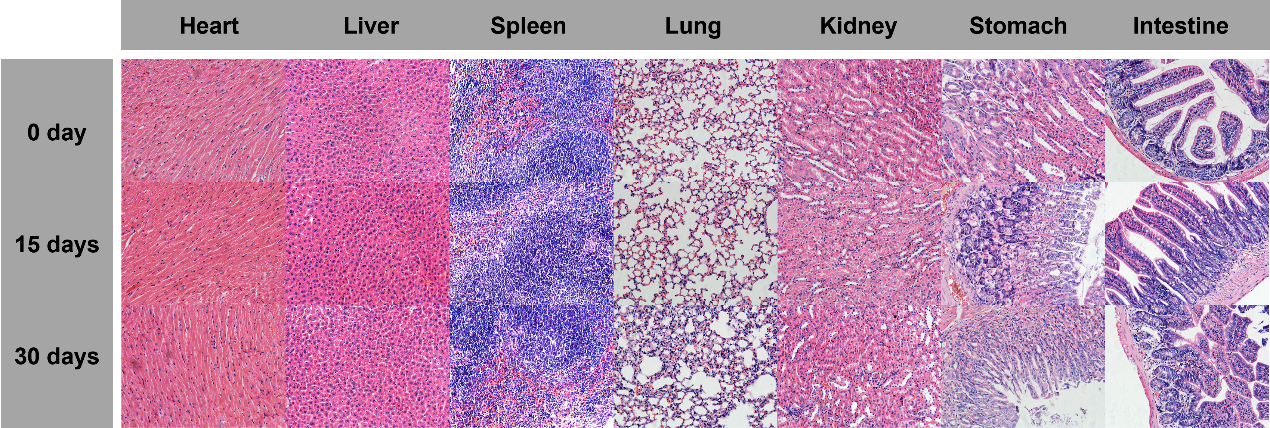


**Figure S1.** Representative hematoxylin−eosin stained histological images of the organs excised after oral administration with mixed solution of HTCC-CA (80 mg/kg) and HPMCP (80 mg/kg) once daily for 15 and 30 days continuously.

**Figure S2.** Fluorescence intensities of the gastrointestinal segments of mice after oral administrations with Cy5-INS/HTCC-CA, Cy5-INS/HTCC/HPMCP and Cy5-INS/HTCC-CA/HPMCP at insulin dose of 30 IU/kg (n = 3). The ordinates are logarithmic coordinates.

**Figure S3.** Relative fluorescence intensity distributions of the gastrointestinal segments of mice after oral administrations with (A) Cy5-INS/HTCC-CA, (B) Cy5-INS/HTCC/HPMCP and (C) Cy5-INS/HTCC-CA/HPMCP (n = 3).

**Figure S4.** Fluorescence intensities of (A) heart, (B) liver, (C) spleen, (D) lung and (E) kidney of mice after oral administrations with Cy5-INS/HTCC-CA, Cy5-INS/HTCC/HPMCP and Cy5-INS/HTCC-CA/HPMCP at insulin dose of 30 IU/kg (n = 3). *P < 0.05.
